# Maintaining Success for Patients With Dilated Cardiomyopathy and Remission of Heart Failure

**DOI:** 10.1016/j.jacbts.2022.03.008

**Published:** 2022-05-23

**Authors:** Brian P. Halliday, John G.F. Cleland

**Affiliations:** a National Heart and Lung Institute, Imperial College London, London, United Kingdom; b Cardiovascular Research Centre, Royal Brompton Hospital, Guy’s and St Thomas’ NHS Trust, London, United Kingdom; c Robertson Centre for Biostatistics and Clinical Trials, Institute of Health and Wellbeing, University of Glasgow, Glasgow, Scotland, United Kingdom; ^d^National Heart and Lung Institute, Imperial College London, London, United Kingdom

**Keywords:** dilated cardiomyopathy, heart failure, HFrEF, HFrEFrem, DCM, dilated cardiomyopathy, HFrEF, heart failure with reduced ejection fraction, HFrEFrem, remission of heart failure with reduced ejection fraction

## Abstract

Remission of heart failure, defined by resolution of symptoms, normalization of left ventricular ejection fraction, and plasma concentrations of natriuretic peptides and by the ability to withdraw diuretic agents without recurrence of congestion is increasingly recognized among patients with dilated cardiomyopathy. Once remission has been achieved, it is unclear which treatments need to be continued long term. The durability of remission and likelihood of relapse are likely to be determined by intrinsic myocardial susceptibility, the persistence or recurrence of any acquired triggers, and current and future myocardial workload. Each of these should be addressed to enable personalized therapy to delay or prevent relapse. Management should be informed by evidence from randomized trials of targeted therapeutic strategies.

## Corresponding Author

**Figure:**
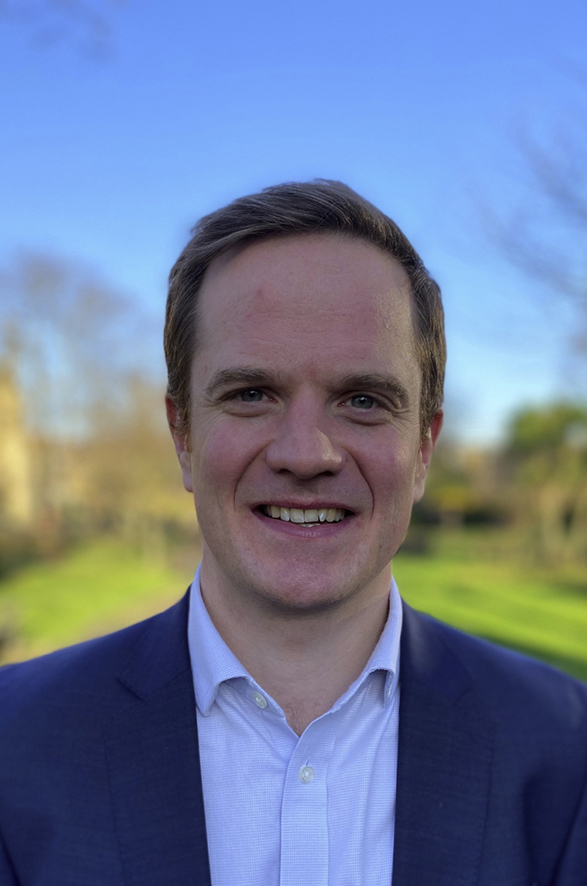

Heart failure with improved ejection fraction, defined as attaining both a ≥10% improvement in left ventricular ejection fraction and to >40%, is increasingly recognized.^1^ Before the use of quadruple disease-modifying therapy, reverse remodeling was observed in a third or more of patients with dilated cardiomyopathy (DCM).^2^ The rate of heart failure with improved ejection fraction will now be even greater with contemporary therapy. There is great diversity within this broad spectrum of recovery, in terms of both normalization of cardiac function and resolution of symptoms, which are not always concordant. A proportion of patients will have remission of heart failure with reduced ejection fraction (HFrEFrem), defined by resolution of symptoms, normalization of left ventricular ejection fraction, and plasma concentrations of natriuretic peptides and by the ability to withdraw diuretic agents without recurrence of congestion. This is more frequent in DCM compared to ischemic heart failure.

Research into the determinants of remission and relapse in DCM provides a huge opportunity to improve our understanding of the biology of heart failure. The range of genetic and acquired causes of DCM introduces complexity but provides the opportunity to understand how different mechanisms interact to influence the likelihood of observing remission and subsequent relapse. DCM may sometimes be attributable to a single cause but will often reflect an interaction between “soil” (innate susceptibility) and “seed” (an external factor or acquired disease). Achieving remission is likely to be highly dependent on the underlying etiology. For example, patients with DCM secondary to a non-missense variant in *LMNA* are unlikely to achieve remission after the onset of heart failure, whereas it is the expected course for many women diagnosed with peripartum cardiomyopathy. For a small proportion of patients, this may reflect true and permanent myocardial recovery, but for many, it will reflect only remission with a risk of recurrence that may be reduced by continuing pharmacologic therapy. The TRED-HF (Therapy Withdrawal in Recovered DCM) trial suggested that many patients in remission will relapse if therapy is completely withdrawn.^3^

## Mechanisms of Relapse and Maintaining Remission

The durability of remission and the likelihood of relapse are likely to be determined by intrinsic myocardial susceptibility and exposure to recurrent myocardial stress. Intrinsic myocardial substrate may be genetic, acquired, or a combination of both. Approximately 20% to 25% of patients will have a rare variant in a gene associated with DCM, most likely a truncating variant in *TTN*.^3^ A greater proportion of patients will have polygenic susceptibility related to common genetic variation. Acquired myocardial disease may be the result of toxins or a previous inflammatory insult that have since been removed or resolved. Extracardiac comorbidities may also contribute to acquired myocardial susceptibility; diabetes mellitus may lead to myocardial fibrosis and microvascular dysfunction, for example. This underlying susceptibility may be unmasked by increased myocardial workload attributable to hypertension or during stressful events such as pregnancy or acute illness. Prolonged tachycardia in the context of atrial arrhythmia is another common cause of decompensation.

**Figure 1:**
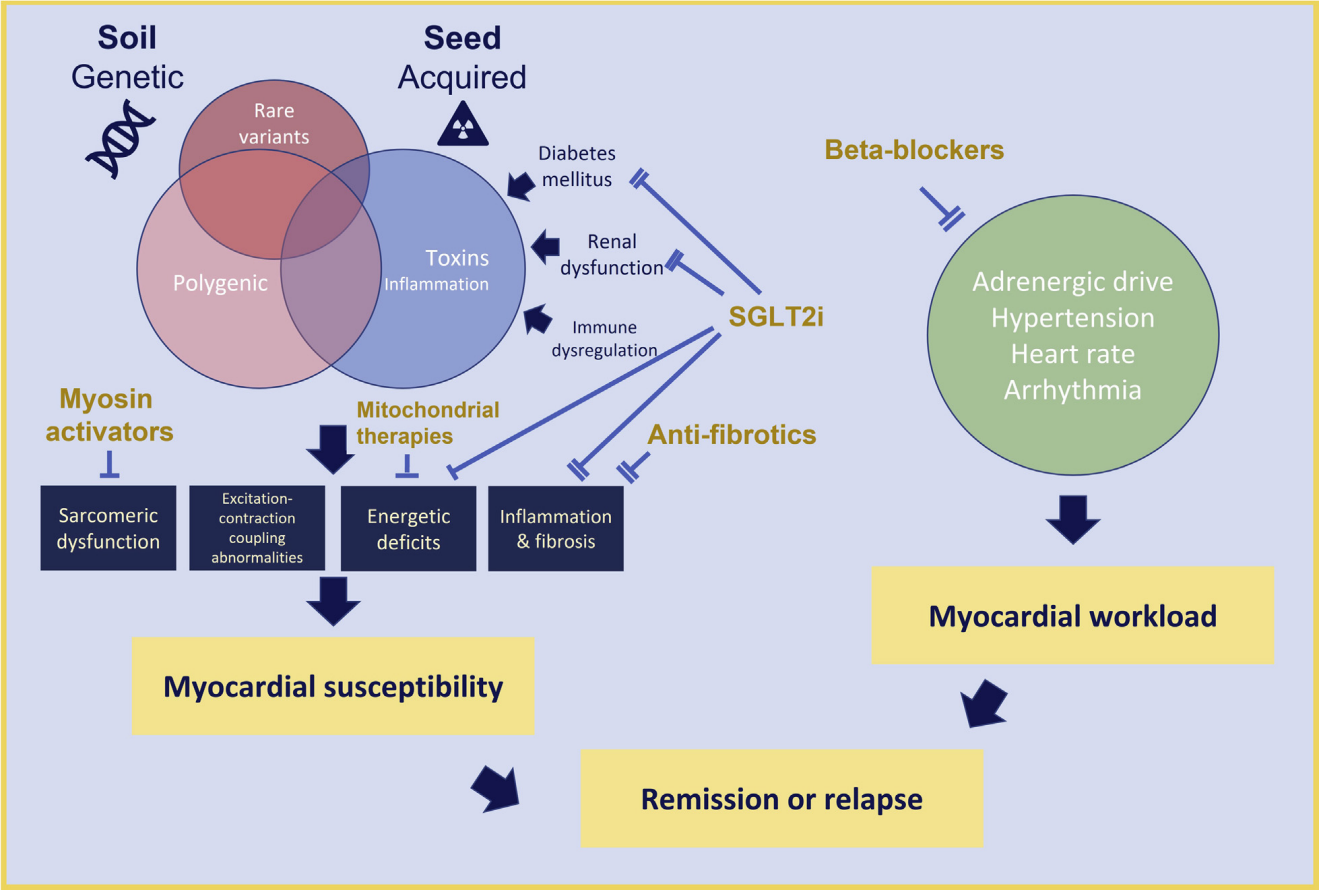
Maintaining Success in Patients With Remission of Heart Failure Developing targeted approaches to maintaining heart failure remission will depend on characterizing the extent of ongoing myocardial susceptibility and balancing this against myocardial workload. Therapies may target different aspects of myocardial susceptibility or myocardial workload.

To achieve and maintain remission, myocardial susceptibility must be managed both by offloading myocardial stress and by removing or preventing acquired triggers. A compensated state is typically achieved with conventional pharmacologic therapy and, occasionally, resynchronization or mechanical unloading. Rises in heart rate and blood pressure appear to precede the development of contractile dysfunction among patients with DCM who relapse during therapy withdrawal,^4^ and it is tempting to speculate that activation of the sympathetic nervous system and a rise in myocardial workload on a vulnerable heart create a self-perpetuating cascade of neurohormonal activation and contractile dysfunction. Preventing autonomic imbalance and overactivity of the sympathetic nervous system leading to increases in heart rate, afterload, and myocardial work may be critical to maintaining remission (Figure 1). Maintaining beta-blocker therapy may therefore be a cornerstone of sustaining remission by reducing myocardial work, particularly during periods of stress.

## Can we Simplify Treatment of Remission Based on Phenotype?

Initiation of disease-modifying therapy is key to improving outcomes among patients with persistent symptomatic HFrEF; more effective therapies and interventions will surely be added in years to come. How to prioritize the initiation of a growing number of therapies at diagnosis is a challenge, and whether patients who achieve remission continue to benefit from all available therapies at the maximum tolerated doses indefinitely is unclear. Whether a simplified pharmacologic regimen based on beta blockers will be successful at maintaining remission once neurohormonal activation has been suppressed, acquired triggers have been removed, and a compensated state has been reached is unknown.

We believe that treatments that target the primary cause of myocardial vulnerability may be the most effective approach to prevent relapse. Measuring the activity of the primary molecular mechanisms that are most likely to cause future decompensation may guide personalized management (Figure 1). Advances in molecular imaging enable the measurement of myocardial energetic deficits and fibrotic activity.^5^ A range of plasma and urinary biomarkers that provide an “omic fingerprint” to assess metabolism, inflammation, fibrosis, and hemodynamics may also be used to identify and manage personalized biotargets. Sodium-glucose cotransporter inhibitors may be effective in preventing heart failure in at-risk populations through a variety of putative effects on many of these disease processes. Given once daily with favorable effects on glycemia and renal function, perhaps this class of drug may represent the second cornerstone of HFrEFrem therapy for many patients.

Our understanding of the role of sarcomeric dysfunction is improving. In the 20% of patients with DCM and improved cardiac function who carry a truncating variant in *TTN*,^3^ sarcomeric dysfunction appears to be an important driver to decompensation. Therapies that augment sarcomeric function may prevent re-expression of disease. Assessment of myocardial mechanics may enable us to predict the consequences of changes in hemodynamics and the likelihood of relapse when patients are exposed to recurrent physiologic stress.

## The Patient with HFrEFrem

When considering whether treatment should be initiated, maintained, or stopped for HFrEFrem, shared decision making between the patient and the health care team informed by evidence is required. Persistent remission and good quality of life should be the aim. What determines quality life will differ among patients, which may affect management decisions. A woman who wishes to conceive after recovering from peripartum cardiomyopathy may consider that stopping therapies that pose a risk to the fetus is the main priority and be prepared to take risks with her own health. Thus, it may be decided to stop an angiotensin receptor–neprilysin inhibitor but continue a beta blocker. The cost of medicines remains prohibitive for some patients. Choosing the most effective combination of medications within their means may not increase the chance of relapse but reduce the financial pressure on them and their family.

Robust evidence, as we have for the initiation of therapies in patients with persistent symptomatic HFrEF, should be sought for phased and selective withdrawal. Although ambitious, it is not infeasible to gather the evidence needed to guide the treatment of HFrEFrem, considering the many millions of patients for whom this question is relevant, now or in the future. Some clinicians may consider investigating the potential de-escalation of therapy unwise. Others may have anecdotal experiences of reducing therapy successfully with a good outcome. It is clear that trials investigating targeted treatment for HFrEFrem should address issues considered priorities by patients in a way that minimizes risk and is informed by current available evidence. The perception and balance between the possible risks and benefits will differ among individual patients based on their own experiences and concerns. Some patients wish to reduce or rationalize medication to improve confidence, reduce side effects, or improve quality of life. Fatigue, cold extremities, dizziness, sleep disturbance, poor concentration, and sleep disturbance are frequently attributed to medications by patients. Clinical trials should not only investigate the risks of therapeutic de-escalation but also the possible benefits and why these occur.

## Future Research

To turn a strategy of targeted, individualized therapy for HFrEFrem from an aspiration into a reality, a series of trials focusing on stratified therapy and based on disease phenotype and mechanism are required. Initial studies may focus on the short- to medium-term effects of the withdrawal of a single agent on myocardial mechanics and remodeling in patients who are perceived to have a low risk of relapse. Early identification of relapse and demonstration that remission can be restored derisks such trials from an individual patient perspective as well as for clinical services and research. These studies may then progress to larger noninferiority studies assessing the effects on longer-term clinical outcomes. Greater understanding of the likelihood of relapse and the mechanistic drivers will guide the decision-making process and enable more personalized selection of the therapies most likely to maintain remission. Large studies will be required to characterize relationships between disease heterogeneity, ongoing treatments, and disease outcomes. These insights will provide a foundation for developing effective and personalized approaches to long-term therapy that are sensitive to patients’ preferences.

## Funding Support and Author Disclosures

Dr Halliday is supported by a British Heart Foundation Intermediate Fellowship (FS/ICRF/21/26019) and by the Rosetrees Trust. Dr Cleland has received research grants from the British Heart Foundation (grant no. RE/18/6/34217); and honoraria from Abbott, Bayer, Bristol Myers Squibb, Medtronic, Moderna, Novartis, Pharmacosmos, Pharma Nord, Servier, and Vifor Pharma, unrelated to this paper.
